# Supplementary material for: Epigenetic age acceleration predicts subject‐specific white matter degeneration in the human brain
Source: Aging Cell. 2024 Nov 28;24(4):e14426. doi: 10.1111/acel.14426 (PMC11984680; doi:10.1111/acel.14426)
Supplement: Supplementary file 1 — Appendix S1. [file ACEL-24-e14426-s002.docx]

**Supplementary Methods**

**Epigenetic Age**

Eight and a half milliliters of whole blood were drawn into a PAXgene Blood DNA Tube (PreAnalytiX, Hombrechtikon, Switzerland). Samples were stored at 20°C for short-term storage (up to 3 months) then transferred to -80°C for long-term storage. DNA was extracted using the PAXgene Blood DNA kit (PreAnalytiX, Hombrechtikon, Switzerland) according to manufacturer instructions. DNA concentration was determined by Quant-iT™ PicoGreen® dsDNA reagent (Thermofisher Scientific, Waltham, MA, USA) per manufacturers instruction. Florescence was detected using a Tecan Infinite M200 Pro microplate reader (Tecan, Switzerland). 500 ng of DNA was bisulfite treated using a Zymo EZ DNA Methylation kit (Zymo Research, Irvine, CA) using the following PCR conditions for Illumina's Infinium Methylation assay (95°C for 30 seconds, 50°C for 60 minutes×16 cycles). DNA methylation was assayed using the Illumina Infinium MethylationEPIC BeadChips. A total of 4μL of bisulfite converted DNA was hybridized to Illumina BeadChips according to the manufacturer's protocols. Samples were denatured and amplified overnight for 20 to 24 hours. After overnight incubation, fragmentation, precipitation, and resuspension of the samples. Samples were then hybridized to EPIC BeadChips for 16 to 24 hours. BeadChips were washed to remove any unhybridized DNA and labeled with nucleotides to extend the primers to the DNA sample. BeadChips were imaged using the Illumina iScan system (Illumina) according to the Infinium HD methylation protocol.

Processing of methylation array data was completed as previously reported^59^. Raw .idat files were read and preprocessed using the minfi R package^45,47^. The data set was preprocessed using noob for background subtraction and dye-bias normalization. All methylation values with detection P>0.01 were set to missing (median sample: 765 probes, range: 319 to 4453), and probes with >1% missing values (n=6,663) were removed from further analysis. All samples were checked and confirmed to ensure that predicted sex matched reported sex. Additionally, samples were checked for excessive missing data (>5%) and unusual cell mixture estimates, which was estimated using the Houseman method as implemented in minfi^48,49^. All samples passed these quality controls. Principal components analysis, as implemented in the shinyMethyl R package, was used to examine batch effects ^46^. The first seven principal components were examined using plots and potential batch effects were tested using linear models. Principal components 3 and 6, which account for 2.38% and 1.65% of total variance respectively, were associated with position on the array (PC3: F_(7, 100)_ = 6.668, p = 1.77e-6, adjusted R^2^ = 0.271; PC6: F_(7, 100)_ = 2.328, p = 0.030, adjusted R^2^ = 0.080). Principal components 1, 4, and 5, which account for 3.63%, 1.89%, and 1.77% of the total variance were associated with bisulfite conversion plate (PC1: F_(1, 106)_ = 9.918, p = 0.002, adjusted R^2^ = 0.077; PC4: F_(1, 100)_ = 34.04, p = 5.932e-8, adjusted R^2^ = 0.236; PC5: F_(1, 100)_ = 31.07, p = 1.91e-7, adjusted R^2^ = 0.219). Principal components 4 and 5, were associated with array (PC4: F_(13, 94)_ = 4.332, p = 1.14e-5, adjusted R^2^ = 0.288; PC5: F_(13, 94)_ = 4.229, p = 1.06e-5, adjusted R^2^ = 0.282). Bisulfite conversion plate and array number were associated with each other, as samples on the same array originated from the same bisulfite conversion plate. Because samples were randomized across plates and arrays, and proportions of variance explained by associated principle components were low, no batch correction method was used. The ewastools R package was used to assess Illumina quality control metrics and call genotypes and donor IDs to ensure the identity of repeated samples from the same individual^50^. All samples passed Illumina quality controls.

To determine assay variability, we included one set of five technical replicates and an additional three sets of two technical replicates. After quality control filters and normalization procedures were applied, the 5,000 CpGs with the most variable M values were used as input for calculating Pearson’s correlation coefficients among all pairwise combinations of samples. Pearson’s correlation of unrelated samples (different individuals) were below 0.8. Pearson’s while correlations of technical replicates ranged from 0.988-0.994, indicating high agreement between technical replicates.

Unnormalized betas were filtered to include CpGs specified by Horvath as necessary for calculation of various clocks. The betas were uploaded to Horvath’s online DNA methylation age calculator (htpps://dnamage.genetics.ucla.edu), which provides measures of Horvath’s multi-tissue age estimator^1^, DNA methylation GrimAge^3^, and cell type abundance. A sample annotation file was included. The options to normalize data and apply advanced analysis were selected. Technical replicates were used to determine measurement error of DNAmAge, the output of Horvath’s multi-tissue age estimator. The absolute difference of DNAmAge between technical replicate pairs was taken, as was the highest absolute difference in the set of five technical replicates. The median of the absolute difference was 2.02 years (range: 0.44-5.73 years).
