# Supplementary material for: Epigenetic age acceleration predicts subject‐specific white matter degeneration in the human brain
Source: Aging Cell. 2024 Nov 28;24(4):e14426. doi: 10.1111/acel.14426 (PMC11984680; doi:10.1111/acel.14426)
Supplement: Supplementary file 2 — Figure S1. [file ACEL-24-e14426-s001.zip › Figure S1 caption.docx]

**Figure S1.** Longitudinal plots from main analysis separated by sex. While men had significantly worse AgeAccelGrim compared to women, the overall distribution of imaging metrics and longitudinal direction were similar between the sexes.
