# Supplementary figures and images for: Epigenetic age acceleration predicts subject‐specific white matter degeneration in the human brain
Source: Aging Cell. 2024 Nov 28;24(4):e14426. doi: 10.1111/acel.14426 (PMC11984680; doi:10.1111/acel.14426)

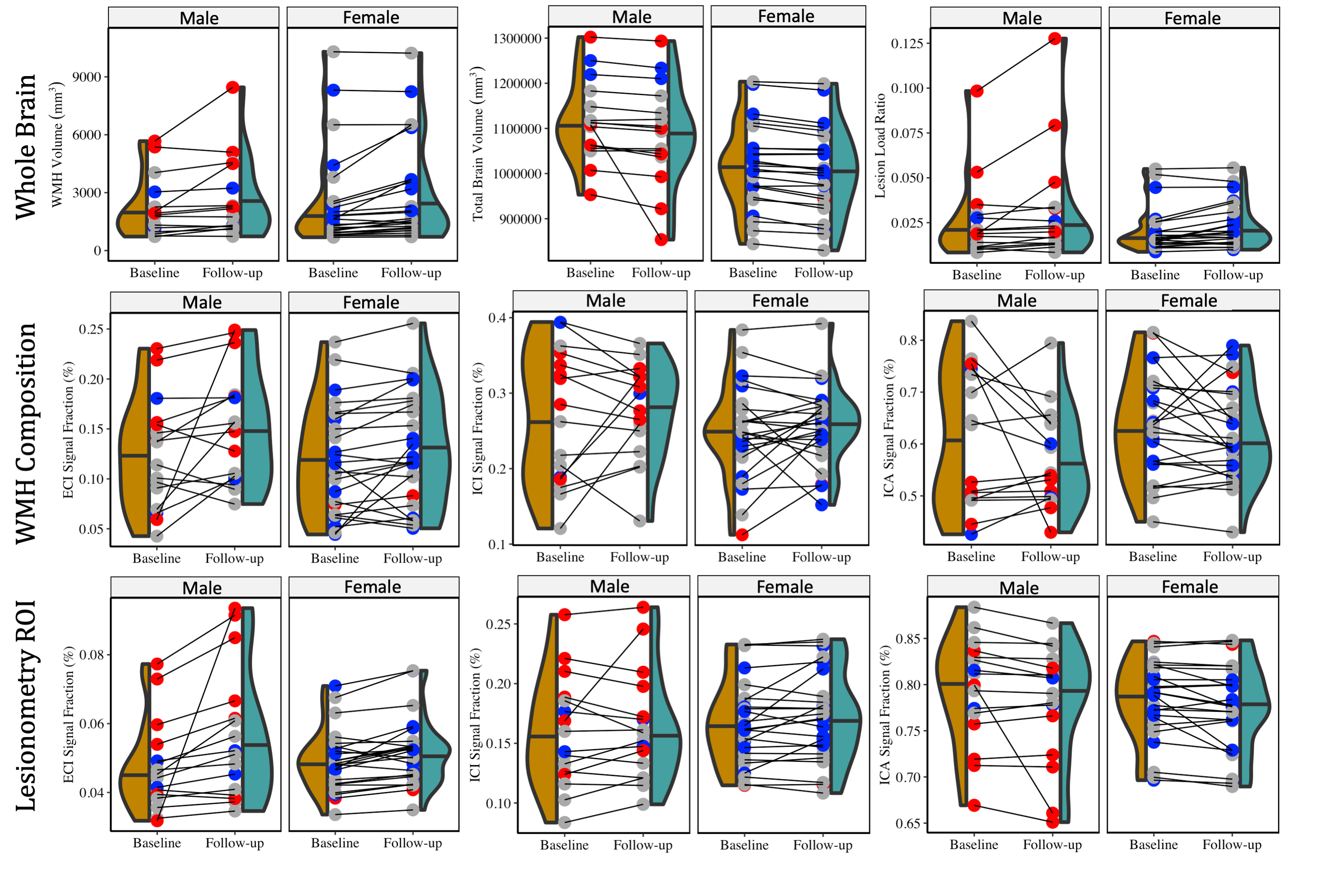

Supplement: Supplementary file 2 — Figure S1. [file ACEL-24-e14426-s001.zip › SupplementaryFigure1.tiff]
